# Pregnancy-related pelvic girdle pain: an update

**DOI:** 10.1186/1741-7015-9-15

**Published:** 2011-02-15

**Authors:** Nikolaos K Kanakaris, Craig S Roberts, Peter V Giannoudis

**Affiliations:** 1 Department of Trauma and Orthopaedics, Leeds Teaching Hospitals NHS Trust, Leeds, UK; 2 Academic Department of Trauma and Orthopaedics, School of Medicine, University of Louisville, Louisville, KY, USA; 3 Academic Department of Trauma and Orthopaedics, School of Medicine, University of Leeds, Leeds Teaching Hospitals NHS Trust, Leeds, UK

## Abstract

A large number of scientists from a wide range of medical and surgical disciplines have reported on the existence and characteristics of the clinical syndrome of pelvic girdle pain during or after pregnancy. This syndrome refers to a musculoskeletal type of persistent pain localised at the anterior and/or posterior aspect of the pelvic ring. The pain may radiate across the hip joint and the thigh bones. The symptoms may begin either during the first trimester of pregnancy, at labour or even during the postpartum period. The physiological processes characterising this clinical entity remain obscure. In this review, the definition and epidemiology, as well as a proposed diagnostic algorithm and treatment options, are presented. Ongoing research is desirable to establish clear management strategies that are based on the pathophysiologic mechanisms responsible for the escalation of the syndrome's symptoms to a fraction of the population of pregnant women.

## Introduction

Pain localised at the pelvic girdle during and after pregnancy has been identified and recorded as an entity since the 4th century BC by Hippocrates. Contemporary medical research since the early 20th century has attempted to clarify the spectrum of the different pathologies that this clinical syndrome represents [1-3].

Despite extensive clinical interest and an increasing number of related publications during the past two decades (Table 1), there is a lack of consensus regarding the incidence, clinical manifestations, treatment algorithms and final outcome of pregnancy-related pelvic girdle pain (PPGP). A large part of the inconsistency can be attributed to the multiplicity and overlapping of the utilised terminology and related definitions (Table 1).

**Table 1:** Existing literature evidence related to pregnancy-related pelvic girdle pain.

Keywords	Number of studies	**Focus of journals,**^**a **^***n***	Era of publications	**Origin of publications**^**b**^
"Pelvic arthropathy"	8 [69,71,100,111,158-161]	Gen Med, 2 [159,161] Obstetr, 5 [69,71,100,111,160] Physioth, 1 [158]	<1985, 6 [69,100,158-161] 1985-1995, 1 [71] 1996-2005, 1 [111] >2005, 0	ESP, 1 [160] GER, 1 [100] RSA, 1 [161] UK, 5 [69,71,111,158,159]
"Osteitis pubis"	9 [1,3,24,99,109,162-165]	Gen Med, 2 [164,165] Gen Surg, 1 [1] Orthop, 1 [109] Radiology, 1 [24] Rheumat, 2 [99,163] Urology, 2 [3,162]	<1985, 5 [1,3,162-164] 1985-1995, 2 [99,165] 1996-2005, 1 [109] >2005, 1 [24]	BRA, 1 [163] FRA, 2 [1,3] POL, 1 [164] TUR, 1 [24] UK, 1 [109] USA, 3 [99,162,165]
"Pelvic insufficiency"	6 [65,66,166-169]	Gen Med, 2 [66,168] Obstetr, 3 [65,166,167] Rheumat, 1 [169]	<1985, 4 [65,66,166,167] 1985-1995, 2 [168,169] 1996-2005, 0 >2005, 0	DEN, 2 [168,169] NED, 1 [66] SWE, 3 [65,166,167]
"Pelvic relaxation pain"	23 [2,61-63,73,75,80,84,106,170-183]	Gen Med, 9 [62,63,75,170,175,178,180,181,183] Obstetr, 12 [2,61,73,80,84,106,172-174,176,177,182] Orthop, 1 [171] Rheumat, 1 [179]	<1985, 9 [2,170-177] 1985-1995, 8 [62,63,75,178-181,183] 1996-2005, 6 [61,73,80,84,106,182] >2005, 0	AUS, 1 [84] CZE, 1 [174] DEN, 5 [61,73,80,106,171] ESP, 1 [170] NOR, 7 [62,63,178-181,183] NZ, 1 [175] TUR, 1 [182] UK, 1 [75] USA, 5 [2,172,173,176,177]
"Pelvic instability"	19 [15,16,64,93,154,155,184-196]	Gen Med, 4 [154,186,193,195] Nursing, 5 [184,185,187-189] Obstetr, 6 [64,93,190-192,196] Orthop, 3 [15,16,155] Psych, 1 [194]	<1985, 7 [155,184-189] 1985-1995, 7 [64,93,154,190-193] 1996-2005, 2 [194,196] >2005, 3 [15,16,195]	AUS, 1 [195] DEN, 7 [155,184-189] NED, 3 [64,93,194] NOR, 4 [190-193] SWE, 1 [196] UK, 1 [16] USA, 2 [15,154]
"Pelvic girdle pain" or "Pelvic pain"	61 [5,9-14,22,34,39,46,50,51,53,77,81,83,86,88,91,92,110,127,133,134,197,17,26,37,38,41,49,107-222]	Anesth, 1 [92] Gen Med, 12 [14,38,49,197,198,201,202,205,212-214,219] Obstetr, 21 [5,10,11,17,22,26,37,39,41,47,88,91,107,204,207,208,210,211,215,217,220] Orthop, 2 [206,218] Physioth, 7 [9,12,46,48,81,134,199] Radiology, 1 [222] Spine, 17 [13,34,40,50,51,53,77,83,86,110,127,133,200,203,209,216,221]	<1985, 1 [204] 1985-1995, 1 [205] 1996-2005, 29 [77,83,86,88,91,107,110,133,134,197-200,206-221] >2005, 30 [5,9-14,17,22,26,34,37-41,46-51,53,81,92,127,201-203,222]	AUS, 3 [38,81,199] CAN, 1 [37] CHN, 1 [53] DEN, 8 [5,86,88,107,209,212,213,219] FRA, 1 [91] IND, 1 [214] IRAN, 1 [34] MEX, 1 [204] NED, 15 [40,41,47-50,77,83,110,134,198,216-218,221] NOR, 7 [14,22,39,46,51,133,200] RSA, 1 [207] SWE, 11 [10-13,17,26,127,201,210,211,220] UK, 4 [92,197,202,208] USA, 6 [9,203,205,206,215,222]
"Posterior pelvic pain"	19 [6,23,52,58,87,89,95,96,101-104,123,223-228]	Gen Med, 2 [226,227] Nursing, 1 [6] Obstetr, 4 [23,52,225,228] Spine, 12 [58,87,89,95,96,101-104,123,223,224]	<1985, 0 1985-1995, 5 [96,104,225-227] 1996-2005, 11 [58,87,89,95,101-103,123,223,224,228] >2005, 3 [6,23,52]	AUS, 1 [103] JAP, 1 [6] NED, 7 [101,102,223,224,226-228] SWE, 6 [58,87,89,95,96,104] UK, 1 [23] USA, 3 [52,123,225]
"Low back pain"	38 [28-32,44,59,68,70,78,79,97,115,125,126,128-132,138,142,144-148,156,157,229-237]	Anesth, 1 [232] Gen Med, 8 [29,32,59,125,128,132,146,233] Obstetr, 15 [28,68,70,126,129-131,145,147,156,230,231,234,236,237] Physioth, 1 [44] Radiology, 2 [115,148] Rheumat, 3 [138,144,229] Spine, 8 [30,31,78,79,97,142,157,235]	<1985, 2 [70,144] 1985-1995, 3 [78,145,146] 1996-2005, 20 [59,68,97,115,126,128-131,138,142,147,148,156,157,229-233] >2005, 13 [28-32,44,79,125,132,234-237]	AUS, 2 [145,236] CAN, 2 [115,125] FIN, 1 [138] GER, 1 [148] HK, 1 [156] NED, 3 [142,234,237] NOR, 4 [70,129,157,229] SWE, 15 [28-32,59,68,79,97,126,130-132,231,232] TAI, 1 [128] TUR, 2 [230,235] UK, 2 [144,233] USA, 4 [44,78,146,147]
"Lumbopelvic pain"	7 [19,33,35,36,43,238,239]	Biomech, 1 [43] Obstetr, 2 [33,36] Physioth, 4 [19,35,238,239]	<1985, 0 1985-1995, 0 1996-2005, 0 >2005, 7 [19,33,35,36,43,238,239]	CAN, 1 [239] NOR, 2 [33,238] SWE, 3 [19,36,43] USA, 1 [35]
"Symphysis pubis dysfunction" or "SPD"	9 [25,76,85,94,105,137,153,240,241]	Anesth, 1 [137] Nursing, 3 [85,105,240] Obstetr, 4 [25,76,153,241] Physioth, 1 [94]	<1985, 0 1985-1995, 0 1996-2005, 6 [85,105,137,153,240,241] >2005, 3 [25,76,94]	NZ, 1 [94] UK, 8 [25,76,85,105,137,153,240,241]
"Pregnancy related pelvic girdle pain" or "PPGP"	10 [4,7,18,20,21,27,60,82,242,243]	Gen Med, 2 [7,242] Obstetr, 2 [82,243] Orthop, 1 [27] Spine, 5 [4,18,20,21,60]	<1985, 0 1985-1995, 0 1996-2005, 1 [60] >2005, 9 [4,7,18,20,21,27,82,242,243]	DEN, 2 [7,243] NED, 5 [4,27,60,82,242] SWE, 3 [18,20,21]
Total, *n *(%)	209	Anesth, 3 (1.4%) Biomech, 1 (0.5%) Gen Med, 43 (20.6%) Gen Surg, 1 (0.5%) Nursing, 9 (4.3%) Obstetr, 74 (35.4%) Orthop, 8 (3.8%) Physioth, 14 (6.7%) Psych, 1 (0.5%) Radiology, 4 (1.9%) Rheumat, 7 (3.3%) Spine, 42 (20.1%) Urology, 2 (1.0%)	<1985, 34 (16.3%) 1985-1995, 29 (13.9%) 1996-2005, 77 (36.8%) >2005, 69 (33.0%)	AUS, 8 (3.8%) BRA, 1 (0.5%) CAN, 4 (1.9%) CHN, 1 (0.5%) CZE, 1 (0.5%) DEN, 24 (11.5%) ESP, 2 (1.0%) FIN, 1 (0.5%) FRA, 3 (1.4%) GER, 2 (1.0%) HK, 1 (0.5%) IND, 1 (0.5%) IRAN, 1 (0.5%), JAP, 1 (0.5%) MEX, 1 (0.5%) NED, 34 (16.3%) NOR, 24 (11.5%) NZ, 2 (1.0%) POL, 1 (0.5%) RSA, 2 (1.0%) SWE, 42 (20.1%) THA, 1 (0.5%) TUR, 4 (1.9%) UK, 23 (11.0%) USA, 24 (11.5%)

The search engine PubMed was utilised for a query (performed 20 January 2010) on the title of the studies, using as keywords^a ^the different terms used in the past to describe the syndrome and as an additional keyword the word "pregnancy" at any of the other fields of the studies. Studies that included more than one different term were inserted once in the table. Underlined are the three most common representatives of each category (that is, "focus of publishing journals" and "origin of publications")

^a^Abbrfeviations of journal subject areas: Anesth, anaesthesiology; Gen Med, general medicine-internal medicine; Gen Surg, general surgery; Obstetr, gynaecology and obstetrics; Orthop; trauma and orthopaedics; Physioth, physiotherapy and rehabilitation; Psych, psychiatry; Rheumat, rheumatology; ^b^Abbreviations of countries: AUS, Australia; BRA, Brazil; CAN, Canada; CHN, China; CZE, Czech Republic; DEN, Denmark; ESP, Spain; FIN, Finland; FRA, France; GER, Germany; HK, Hong Kong; IND, India; JAP, Japan; MEX, Mexico; NED, The Netherlands; NOR, Norway; NZ, New Zealand; POL, Poland; RSA, South Africa; SWE, Sweden; THA, Thailand; TUR, Turkey; UK, United Kingdom; USA, United States of America.

The scientific and clinical implications of PPGP require the multidisciplinary interaction of a wide number of health-related specialties, including obstetrics and gynaecology, general medicine, orthopaedic surgery, physiotherapy, rheumatology and clinical psychiatry (Table 1). This important parameter is another strong factor that affects the discrepancy and fragmentation of the reported data between different journals and scientists not directly communicating with each other.

Lately, efforts to establish guidelines and accurate definitions of the manifestations of this clinical syndrome have been ongoing and offer the basis for further international research [4]. Following the publication of the European Guidelines in 2005 [4], the authors of 49 subsequent clinical studies [5-53] incorporated, to a degree, the recommended methodology. In parallel, the patient community in the modern era of widespread interactive communications has launched a number of websites and forums focusing on the problem and seeking advice and guidance [54-57].

**Figure 1:**
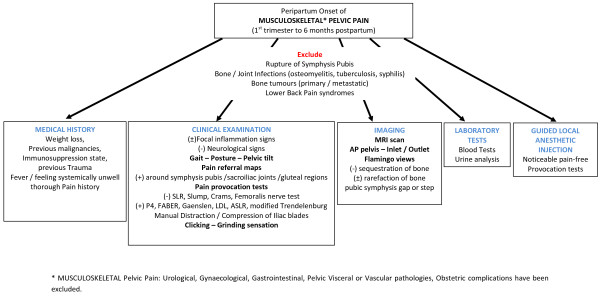
**Diagnostic algorithm of peripartum pelvic girdle pain**.

The aim of this minireview article is to present in a comprehensive manner the existing consensus regarding the diagnosis, management and prognosis of PPGP. The PubMed search engine was used to set a query on 20 January 2010 with the keywords "pelvic arthropathy" OR "osteitis pubis" OR "pelvic insufficiency" OR "pelvic pain" OR "pelvic instability" OR "pelvic girdle pain" OR "posterior pelvic pain" OR "low back pain" OR "lumbopelvic pain" OR "symphysis pubis dysfunction" in the title, as well as the term "pregnancy" in any of the search fields of the publications. Whenever additional studies were identified from the references of the retrieved publications, they were also included in this review. In total, 209 studies from 1923 to today are presented in this review according to the terminology that was used by the authors, the decade of publication and the origin of the research (Table 1). Further attention and value were given to those of the 209 studies that represent the highest level of evidence, derived their conclusions from large samples (>30 cases), and took into account contemporary definitions and diagnostic and treatment methodologies. These studies are the ones mostly commented on and presented in this article, as well as in the proposed algorithm of patient management (Figure 1).

### Definition

Many terms have been used to describe PPGP syndrome on the basis of causative hypotheses (pelvic joint arthropathy, relaxation, insufficiency, instability), presenting symptoms (pelvic pain, and/or low-back pain, pelvic joint pain) or related topography (posterior pelvic pain, osteitis pubis, symphyseal pelvic dysfunction, low-back pain) (Table 1).

All of these attempts to define the problem have been unsuccessful either because they narrowed the spectrum of this pain syndrome or because they confused its nature by blending it with the syndrome of chronic low-back lumbar pain. There is an existing consensus [58,59] that pregnancy-related low-back pain is a distinct entity that needs to be excluded before the diagnosis of PPGP is made.

While the responsible pathophysiological mechanisms remain obscure, this clinical syndrome is best defined descriptively by its presentation and topography. With regard to its onset, it has been associated with symptoms beginning between the first trimester, at labour or even during the postpartum period. Thus, terms limiting PPGP to a certain phase pregnancy appear insufficient to cover the whole spectrum of the clinical problem. With regard to this concept, the European Guidelines [60] are based on the musculoskeletal type of the resulting pain (excluding gynaecological and/or urological causative pathologies) localised from the level of the posterior iliac crest and the gluteal fold over the anterior and posterior elements of the bony pelvis. In 2005, the term pregnancy-related pelvic girdle pain, or PPGP, was introduced and appears to be the most accurate compared with previous definitions.

### Aetiology

The exact mechanisms that lead to the development of PPGP remain uncertain. A variety of approaches have been proposed that suggest hormonal [61-64], biomechanical [65,66], traumatic [67], metabolic [68], genetic [69] and degenerative [70,71] etiologic implications.

On the basis of all of these hypotheses, the accumulated evidence advocates in favour of a multifactorial condition during pregnancy and postpartum. The effect of the levels of relaxin and progesterone to the pelvic girdle ligaments is established [72]; however, no consensus of its association with the symptoms of PPGP has been reached [73-75]. This discrepancy can be attributed mostly to methodological differences [76], as well as to the presence of unspecified cofactors altering the clinical presentation. The biomechanical theory and its advocates [15] have highlighted the separation of the pubic symphysis (≥10 mm) as an important threshold. However, this was not proven to be consistent and does not apply to patients with symptoms mostly localised at the posterior pelvic girdle. Moreover, other mechanical theories [77,78] based on body habitus and lumbar spine stance, as well as foetal size and weight, have also been proven incompatible with all the cases. The role of genetics is still largely unknown, and current knowledge is based on epidemiological indications between first-degree relatives [79-81].

### Risk factors

Among a large number of potential factors, those of strenuous work (twisting and bending the back several times per hour), a history of low-back pain, pelvic girdle pain or previous trauma to the bony pelvis were identified [4,5,53,80,82,83] as being strongly related to PPGP. Conversely, in the same epidemiologic observational studies, factors such as the time from previous pregnancies, smoking habits, use of contraception, epidural anaesthesia, maternal ethnicity, body mass index, number of previous pregnancies, bone density, foetal weight and age were not linked with increased risk of PPGP development.

### Incidence

Among all the relevant studies, the incidence of PPGP ranges from 4% to 76.4% depending on the definition used, the diagnostic means utilised (for example, patient history, pain questionnaires, clinical tests) and the design of the studies (retrospective or prospective).

As reported by Wu *et al*. [83], on average, doctors' files verify the syndrome in about 20% fewer cases than patients' reports. The apparent geographical variation of reported PPGP incidence and severity, with higher rates in Scandinavian countries [80,84] and the Netherlands [47,83], should be attributed to the increased awareness regarding this condition by healthcare providers and the public [76,85]. However, the reported cases are spread among a wide variety of countries (Table 1) and across all continents, indicating that PPGP is a universal problem.

Using the definition described above and including only prospectively designed studies of large series of patients with objectively verified symptoms, the prevalence of PPGP is between 16% and 25% [4,80,83,86-88]. Over the same large samples of pregnant women, the clinically persistent PPGP from the postpartum stage to 2 years after childbirth has a reported incidence of 5% to 8.5% [83,86,88,89].

### Differential diagnosis

The PPGP diagnosis should be considered after the exclusion of painful visceral pathologies of the pelvis (urogenital, gastrointestinal), lower-back pain syndromes (lumbar disc lesion/prolapsed, radiculopathies, spondylolisthesis, rheumatism, sciatica, spinal stenosis or lumbar spine arthritis), bone or soft tissue infections (typical or atypical such as tuberculosis or syphilitic lesions of pubis), urinary tract infections, femoral vein thrombosis, obstetric complications (preterm labour, abruption, round ligament pain, chorioamnionitis), rupture of symphysis pubis, and bone or soft tissue tumours [13,19,37,90,91].

A thorough medical history, physical examination and appropriate laboratory tests should always be performed to successfully reach the diagnosis of PPGP. Obviously, a multidisciplinary approach and consultation may be needed, as this syndrome expands to a wide field of anatomically related medical specialties [4,6,24,40,60]. An algorithm of the necessary diagnostic workup is presented in Figure 1.

### Presentation, classification and diagnosis

PPGP, as defined previously, has been associated with pain (stabbing, dull, shooting, burning) located at the general area of pelvic girdle, either posteriorly close to the sacroiliac joints and extending to the gluteal area or anteriorly to the vicinity of the symphysis pubis. It may radiate to the groin, perineum or posterior thigh, lacking a typical nerve root distribution. A precise localisation of the pain is often impossible and may also change during the course of the pregnancy [74,92].

Current classification systems of PPGP are based on pain localisation [86,92]. They include five subtypes: (1) type 1 or "pelvic girdle syndrome," comprising symptoms of anterior and posterior pelvic girdle, symphysis pubis and bilateral sacroiliac joints; (2) type 2 or "double-sided sacroiliac syndrome," comprising symptoms of the posterior pelvic girdle and bilateral sacroiliac joints; (3) type 3 or "single-sided sacroiliac syndrome," comprising symptoms of the posterior pelvic girdle and unilateral sacroiliac joint; (4) type 4 or "symphysiolysis," comprising symptoms of the anterior pelvic girdle and pubic symphysis; and (5) type 5 or "miscellaneous," comprising inconsistent findings of the pelvic girdle.

The onset of PPGP varies significantly and has been recorded at stages between the end of the first trimester to the first month postdelivery, including the labour stage [76,78,93,94]. It may be insidious or sudden. In general, postpartum pain may be milder than that during pregnancy. A general consensus exists regarding a peak of symptoms closer to the third trimester between the 24th and 36th weeks of pregnancy [76,94]. In the majority of cases (up to 93%), PPGP settles and spontaneously disappears after the sixth month postpartum. In the rest of the cases, it persists, acquiring a chronic character.

Several authors [4,50,83] have recommended that a careful recording of the pain history of the patient suspected of having PPGP contributes significantly to a successful diagnosis. Characteristics such as exacerbations related to a change of position from sitting to standing or during prolonged sitting or standing, during sexual intercourse, and increased intra-abdominal pressure (coughing, sneezing, micturition, defecation) should be explored. On the basis of the medical history, changes and significant difficulties in performing activities of daily living are usually apparent. History combined with the localisation of the pain, with the addition of pain referral maps [95], can differentiate lower-back pain syndromes, sciatica, visceral or vascular origin syndromes from PPGP.

PPGP pain intensity is repeatedly reported [83,89,96,97] to be around 50 to 60 mm of the visual analogue scale (VAS), ranging significantly, however, throughout the duration of the syndrome from bearable to very serious for the 8% of severely disabled women. Wu *et al*. [53] described a higher correlation of the resultant disability to the increased "fear of movement" and less to the degree of pain itself.

Alteration of gait patterns has also been associated with the syndrome regarding the inability of these patients to cover long distances or a temporary "catching" sensation or clicking on hip flexion, located mostly anteriorly or unilaterally posteriorly. The gait coordination of these patients is distinctly characterised by slower walking velocity, an increase in the amplitude of the horizontal rotation of the pelvis to the thorax and a reduced relative phase between these rotations, which differentiate PPGP patients from those with lower-back pain and healthy pregnant women [50,53,98].

Tenderness to deep palpation of the suprapubic and sacroiliac area along the course of the long posterior sacroiliac and sacrotuberous ligaments, as well as a palpable step of the pubic symphysis joint, may be evident. Signs of local inflammation (erythema, oedema, warmth) may exist in a small percentage of the cases [99,100].

A wide variety of clinical examinations have been evaluated regarding their usefulness in the assessment and differential diagnosis of PPGP. Earlier studies were more focused on deep palpation and radiologic findings, while lately the weight of diagnosis has shifted toward the cumulative results of specific pain provocation tests [4,6,101-104]. For the posterior elements of the pelvic girdle and the sacroiliac joints, the most reliable examinations are the posterior pelvic pain provocation test (P4/thigh thrust), the Patrick's FABER (flexion, abduction, external rotation at the hip), the active straight leg raise (ASLR), the long dorsal ligament and the Gaenslen tests [4,6,101-104]. With regard to the pubic symphysis, the diagnosis is based mostly on deep palpation and the modified Trendelenburg test [25,84,105].

Because most of these tests have a proven high specificity but lower sensitivity, there appears to be a consensus for the combined use of all of these tests to minimise false-negative results. Leadbetter *et al*. [25] described a scoring system to guide clinicians in screening the general pregnant patient population. In that system, they included five essential symptoms: pain of the pubic symphysis on walking, while standing on one leg, while climbing stairs, or while turning over in bed, as well as a history of damage to the pelvis or the lumbosacral area.

Laboratory blood tests are usually normal, with a nonspecific mild elevation of the acute phase reactants (C-reactive protein, erythrocyte sedimentation rate) in a number of cases. However, for reasons related to differential diagnosis, most authors report acquiring a complete blood count, biochemistry and urine analysis [75,106,107].

Radiological investigations have a more essential role in the evaluation of the PPGP syndrome. Standard anteroposterior, inlet and outlet pelvic films are used to measure the degree of symphyseal separation and to identify cortical sclerosis, spurring or rarefaction. The use of single-limb stance anteroposterior or flamingo views delineates more subtle cases of pubic symphysis separation and appears useful in quantifying the degree of pelvic girdle instability [108]. The detection of a step-off of more than 2 or 7 mm at the standard anteroposterior or flamingo views, respectively, is considered by some authors as a threshold of pelvic instability [109]. However, no direct association of the extent of the separation or of the radiologic irregularities to the severity of PPGP was identified in a number of studies [15,109-114]. Computed tomography (CT) scanning has also been performed by some authors, mainly for differential diagnosis [115-117]. However, according to the recent recommendations of the European PPGP research group [4], conventional radiography, CT scans and scintigraphy are inadequately supported for their use in rendering a PPGP diagnosis.

These imaging techniques are usually limited to postpartum females because of the hazard of exposing the foetus to ionising radiation. A magnetic resonance imaging (MRI) scan is suggested during pregnancy, offering additional advantages of increased resolution and its superiority in allowing visualisation of soft tissue and marrow reactions [24,118-120]. In addition, according the European guidelines, the MRI scan is recommended for the differential diagnosis of PPGP in all its stages [4].

Transvaginal/transperineal ultrasonography has also been advocated for the diagnosis and monitoring of the progress of pubic symphysis PPGP, with the limitation of being a user-dependent examination [42,111,113,121,122].

Last, guided local anaesthetic injections to the sacroiliac or pubic symphysis joint and the resulting pain relief during previous positive provocation tests offer significant diagnostic specificity, reaching 100%, but reflect only intra-articular pathologies. PPGP related to extra-articular pathologies may be unaffected (that is, strain of the superficial long sacroiliac joint ligament) [103,123].

### Management

Because of the large heterogeneity of the published studies and the inconsistent quality of the reviewed articles (ranging from large, randomised, controlled trials to uncontrolled case series and case reports), no strong comparative evidence regarding the utilised methods of treatment is possible. Management of the PPGP syndrome as reported during the past few decades involves a variety of clinicians and specialities, as well as a combined interdisciplinary approach.

**Figure 2:**
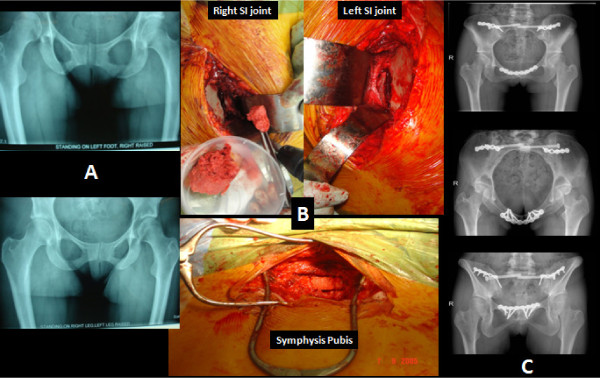
**Female patient 38 years of age with persistent type 1 **[86]**peripartum pelvic girdle pain (PPGP) that was resistant to nonoperative means of therapy**. The patient underwent triple pelvic joint fusion 2 years after delivery of her second child. **(A) **Stork views and radiological evidence of pubic symphysis instability. **(B) **Intraoperative images of bilateral sacroiliac joints after debridement at the time of grafting and of the pubic symphysis after debridement and application of autologous tricortical bone graft. **(C) **Radiological confirmation (anteroposterior, inlet and outlet views) of healing of all fusion sites 7 months postoperatively. The patient mobilized independently, experienced significant pain relief and returned to work.

Before labour, the available options for its management are limited by the presence and the potential hazards to the foetus. Also, the majority of symptomatic patients appear to recover gradually after the first few months postdelivery. For these reasons, a proposed algorithm of management should differentiate between pre- and postpartum cases (Figure 2). Bed rest and symptomatic care appear to be the mainstay of PPGP therapy, at least at its initial stages [4,12,47,85,124,125]. Water gymnastics [126] and pelvic tilt exercises [58,127,128], with avoidance of maladaptive movements [129], as well as acupuncture [130,131] and physical fitness exercises at early pregnancy [132] have been identified as beneficial on the basis of the level of reported pain and have been associated with a decrease of the sick leave taken by prepartum patients.

Regarding the cases that remain symptomatic postdelivery, it has been shown that treatment based on specific stabilising exercises offers significant advantages over pain management, functional recovery and generalised health-related quality of life and physical status [133,134]. Individually tailored, supervised physical therapy is reported to be more effective than general back and/or pelvic pain therapies [46,58,104].

Pain relief drug therapies have been evaluated extensively in the literature. The reported consensus is that paracetamol, although safe for use in the pregnant population, is considered inadequate on its own for the PPGP levels of pain. Nonsteroidal anti-inflammatory drugs (NSAIDs) have a better pain relief effect but are linked to foetal malformations or pregnancy complications [135]. Luckily, the severity of PPGP symptoms also peaks at the end stages of pregnancy, allowing for NSAID use then or mostly postpartum. Opioids are strictly restricted in the prepartum cases, as well as among lactating females [92]. In a few small series [23,136,137], the use of epidural analgesia has been reported with good results, delivered either in a single shot or in extended administration during periods of pain exacerbation. In all cases, it should be considered as a temporary method of pain relief until delivery.

The use of guided injections of local anaesthetics with corticosteroids was tested therapeutically in cases of evident arthritis of the pelvic joints [138,139]. In several studies [16,140-142], they were used preoperatively to justify surgery for fusion of the painful pelvic joint or triple fusion of all pelvic articulations. The methods of guidance vary between fluoroscopy, CT and MRI scans, offering targeted administration of chemicals to the degenerative joints without specific evidence of the advantages of one chemical over the others.

A limited number of studies have evaluated the efficacy of antenatal back care education and supplemental therapies such as massage [143], local application of heat and/or cold [46], modified back school classes [96,144], special pillows [145], sacroiliac joint manipulation and mobilisation [146], pelvic belts [110,147], radiofrequency denervation of the pain receptors of the sacroiliac joints [148] and transcutaneous electrical nerve stimulation [92,149], with inconclusive or unconvincing results. A generalised recommendation in the experimental use of some of these methods (cushions and pillows, early patient education and general fitness exercise programs, walking aids and/or wheelchairs) was recently suggested [92] on the basis of the potential beneficial psychophysiological effect at least to a subgroup of the PPGP population and the apparent safety of these noninvasive approaches.

The labour of a pregnant woman with established PPGP syndrome appears to be the phase less investigated with regard to its relationship to the persistence of the symptoms postdelivery. However, there appears to be a consensus regarding minimal stress on the pelvic girdle, avoidance of abduction of the hips over the prespinal/epidural anaesthesia comfort arc of the particular patient and minimisation of the duration of the lithotomy position ("all-four" position or lateral positions should be used instead) [150-152]. Caesarean section does not appear to offer any particular advantages to women with established PPGP syndrome, except for those at the worst extreme, whereas the mere positioning for vaginal delivery is impossible [31,92,151]. Early induction of labour or elective caesarean section is advocated by a few of the authors [85,153] in the most severe cases, but these options are still supported by limited evidence.

Pelvic fusion surgery has been evaluated in a number of case series studies [16,140-142] and in general represents an end-stage procedure following the failure of nonoperative means and the persistence of debilitating symptoms. A number of authors [154,155] have advocated in favour of a staged approach, with the application of an external fixator as a temporary stabilisation device serving as an indicator of the potential relief of symptoms if mechanical instability is the main causative factor. Most of these cohort studies represent the experience of tertiary referral centres and report on fusion surgery of one or all three of the pelvic girdle joints (Figure 2). According to the European guidelines [4], the surgical option should be offered as part of a comprehensive management protocol and mostly as an end-stage alternative used by specialist surgeons.

### Prognosis

The reported outcomes for patients with PPGP appear to be universally good in the vast majority of prepartum cases. The syndrome is described mostly as a self-limiting condition in which symptoms settle in 93% of the patients within the first 3 months postdelivery. By the first year postdelivery, only 1% to 2% of patients report the persistence of pain. These cases are mostly those patients who had very intense symptoms during the pregnancy period. As reported by Albert *et al*. [88], 79% of those with severe PPGP symptoms are asymptomatic 2 years postdelivery.

Among several related studies [7,8,21,30,41,87,88,156,157], certain risk factors for a worse prognosis have been identified. They are based on the patient's history and demographic, psychosocial and socioeconomic characteristics as well as the intensity of PPGP symptoms. A high number of simultaneously positive provocation diagnostic tests, a lower index of mobility, lack of education and/or unskilled work history, multiparity, prolonged duration of labour, age >29 years, higher pain intensity (VAS score >6), onset of pain at early gestation, combined lumbar and pelvic pain in pregnancy and localisation of pain in more than one of the pelvic joints are all included among these adverse prognostic factors. A positive ASLR test and belief in improvements have both been regarded as important independent factors by Vollestad and Stuge in their recent publication [51].

Recurrence of PPGP is commonly reported (41% to 77%), either with a subsequent pregnancy or related to the menstrual cycle [76,77,80]. The exact incidence of recurrence, as well as its related risk factors or the role of preventive measures, is unknown. In the majority of the recorded pregnancy relapses of PPGP, the syndrome reappears in a more severe form [84,85].

## Conclusion

Contemporary clinical awareness of the PPGP syndrome appears to be increasing because of increased public awareness and the interaction of scientists from different medical specialties. Recently introduced definitions and proposed guidelines on PPGP diagnosis and management represent significant improvements, setting the basis for future comprehensive research on this multifactorial pain syndrome. Different treatment modalities and disease-specific outcome measures need to be investigated in multicentre, randomised clinical trials following the previous initiative of the Research Directorate of the European Commission [4].

## Competing interests

The authors declare no competing interests, the absence of any funding related to this article, no ethical approval was applicable, and there are no guarantors or acknowledgements.

## Authors' contributions

NKK participated at the design of the article, the acquisition of the data from the reviewed articles, their analysis and interpretation and the initial drafting of the manuscript. CSR assisted with the preparation of the final manuscript. PVG conceived the article and participated in its design and the final revision of the manuscript.

## Pre-publication history

The pre-publication history for this paper can be accessed here:

http://www.biomedcentral.com/1741-7015/9/15/prepub
